# Dynamics of outcome after aneurysmal subarachnoid hemorrhage

**DOI:** 10.18632/aging.103069

**Published:** 2020-04-20

**Authors:** Alexander Hammer, Gholamreza Ranaie, Eduard Yakubov, Frank Erbguth, Markus Holtmannspoetter, Hans-Herbert Steiner, Hendrik Janssen

**Affiliations:** 1 Department of Neurosurgery, Paracelsus Medical University, Nuremberg 90471, Bavaria, Germany; 2 Department of Neurology, Paracelsus Medical University, Nuremberg 90471, Bavaria, Germany; 3 Department of Neuroradiology, Nuremberg General Hospital, Nuremberg 90471, Bavaria, Germany; 4 Department of Neuroradiology, Ingolstadt General Hospital, Ingolstadt 85049, Bavaria, Germany

**Keywords:** intracranial aneurysm, subarachnoid hemorrhage, outcome, aging, stroke

## Abstract

In this observational study, we analyzed and described the dynamics of the outcome after aneurysmal subarachnoid hemorrhage (SAH) in a collective of 203 cases. We detected a significant improvement of the mean aggregate modified Rankin Score (mRS) in every time interval from discharge to 6 months and up to 1 year. Every forth to fifth patient with potential of recovery (mRS 1-5) at discharge improved by 1 mRS point in the time interval from 6 month to 1 year (22.6%). Patients with mRS 3 at discharge had a remarkable late recovery rate (73.3%, p = 0.000085). Multivariate analysis revealed age ≤ 65 years (odds ratio 4.93; p = 0.0045) and “World Federation of Neurological Surgeons” (WFNS) grades I and II (odds ratio 4.77; p = 0.0077) as significant predictors of early improvement (discharge to 6 months). Absence of a shunting procedure (odds ratio 8.32; p = 0.0049) was a significant predictor of late improvement (6 months to 1 year), but not age ≤ 65 years (p = 0.54) and WFNS grades I and II (p = 0.92). Thus, late recovery (6 month to 1 year) is significant and independent from age and WFNS grade.

## INTRODUCTION

Beyond the first 6 months after subarachnoid hemorrhage (SAH), there is a controversy as to whether it is reasonable to conduct studies at later time points for outcome determination, such as 1 year and above - not only regarding SAH, but for stroke itself [1–5]. There is growing evidence that these later phases of rehabilitation should not be underestimated [1, 4, 6, 7]. Regarding the degrees of recovery at different time points and their comparisons, the data is lacking and the available studies are at least partly retrospective, with low patient numbers, presenting univariate statistics or presenting only poor-grade patients after suffering from SAH [1, 4]. The aim of this study was to have a closer look at this dynamic process of rehabilitation of patients suffering from SAH. In these terms, we used a complete collective of SAH patients comprising “World Federation of Neurological Surgeons” (WFNS) grades I to V and used our prospective collected outcome data at discharge, at 6 months, and at 1 year, together with our baseline data, data of comorbidities, and intensive care complications, in order to detect significant changes of outcome over time and to detect significant predictors of outcome improvement after 6 months and after 1 year.

## RESULTS

We used the data from our observational study cohort from the years 2012–2017 with 203 patients. The baseline data for this collective were published previously [9]. For the analysis of the predictors of improvement, 157 patients were available for the univariate analysis in terms of a chi-square test. We dichotomized the tested variables in order to create the crosstabs. The significant variables data in terms of frequency and percentage can be seen in Table 1.

**Table 1:** Categorized baseline data (n = 157) and average time in ICU.

**Variables**	**n**	**Percentage**
Age ≤ 65 y	127	80.9%
WFNS I–II vs III–V	97 vs 60	61.8% vs 38.2%
Discharge mRS (1–2 vs 3–5)	83 vs 74	52.9% vs 47.1%
Early cerebral ischemia	26	16.6%
Delayed cerebral ischemia	30	19.1%
History of smoking	65	41.4%
Pneumonia	66	42.0%
Hydrocephalus	73	46.5%
Shunting procedure	29	18.5%
Tracheostomy	53	33.8%
Decompression	31	19.7%
Time in ICU (≤ 3 weeks vs > 3 weeks)	100 vs 57	63.7% vs 36.3%
	**Mean**	**Standard deviation**
Average time in days in ICU	19.43 (4–76 days)	+/- 9.8

### Modified rankin scale data analysis over time

**Figure 1:**
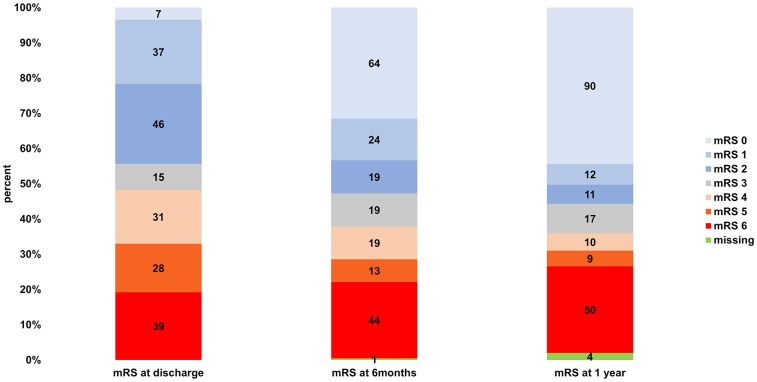
**Stratified mRSs at discharge, 6 months, and 1 year.**

**Figure 2:**
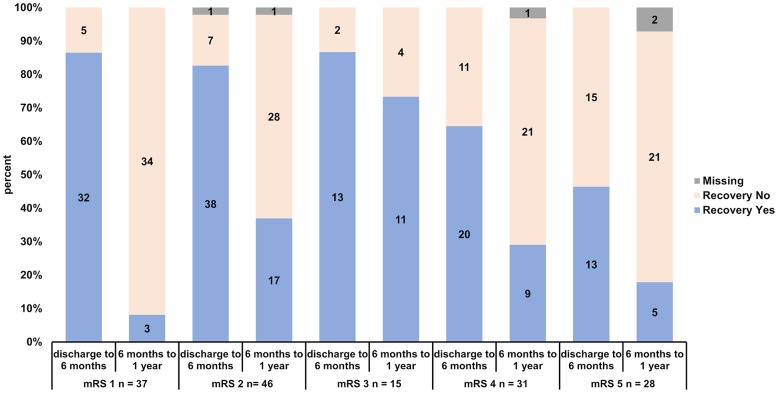
**Recovery rates of patients stratified according to initial mRS (mRS 1, 2, 3, 4 and 5) and according to early (discharge to 6 months) and late recovery (6 months to 1 year).**

From discharge to 6 months, 116 of 202 patients (57.4%) improved (-1 mRS point), and 86 (42.6%) had the same or a higher mRS. In the time interval from 6 months to 1 year, 45 of 199 (22.6%) improved regarding the mRS, and 154 of 199 (77.4%) did not. The mean aggregate mRSs at discharge, 6 months, and 1 year can be seen in Table 2. The paired samples t-test showed a significant improvement on all tested time intervals. Within the available pairs (n = 202; 1 lost to follow-up at 6 months), the aggregate mRS at discharge was 3.32 and the aggregate mRS at 6 months was 2.59. The improvement from discharge to the 6-month follow-up was significant (p < 0.0001). There was also a significant improvement regarding the pairing of the mean aggregate mRSs from 6 months to 1 year (n = 199; 1 lost to follow-up at 6 months and 4 lost to follow-up at 1 year; mRS at 6 months of 2.58 vs mRS at 1 year of 2.36; p = 0.00077), although the difference was considerably smaller (mRS improvement of 0.73 at 6 months vs 0.22 at 1 year). The total significant mRS improvement over 1 year was 0.94 within the 199 available pairs (4 lost to follow-up at 1 year); the aggregate mRS at discharge was 3.30 and the aggregate mRS at 1 year was 2.36 (p < 0.0001). The stratified mRSs at discharge, 6 months, and 1 year are illustrated in Figure 1. A graphic shift analysis for the initial mRS values describing the recovery 6 months and 1 year after discharge can be seen in Figure 2.

**Table 2:** Mean aggregate mRSs at discharge, 6 months, and 1 year.

**Time interval**	**Compared mRS scores +/- SD**	**p-value**	**n**
mRS at discharge vs mRS at 6 months	3.32 +/- 1.91 vs 2.59 +/- 2.37	<0.0001	202
mRS at 6 months vs mRS at 1 year	2.58 +/- 2.38 vs 2.36 +/- 2.55	0.00077	199
mRS at discharge vs mRS at 1 year	3.30 +/- 1.91 vs 2.36 +/- 2.55	<0.0001	199

Paired samples t-test validated significant improvement on every tested time interval.

### Predictors of early improvement in the univariate analysis

We used the dichotomized data of 156 patients (n = 157; 1 missing at 6-month follow up) for the chi-square test regarding the impact on the improvement of the mRS. The following variables were significant predictors of early improvement (discharge to 6 months) in the univariate analysis: age ≤ 65 y (p = 0.0020), WFNS I or II (p < 0.0001), discharge mRS score 1–2 (p = 0.00092), absence of delayed cerebral ischemia (p = 0.045), no history of smoking (p = 0.035), absence of pneumonia (p = 0.0027), absence of hydrocephalus (p = 0.0075), absence of tracheostomy (p = 0.0041), no decompression (p = 0.020), and time in ICU ≤ 3 weeks (p = 0.015) (see Table 3). The following tested variables did not reach significance: intervention (clipping vs. coiling), sex, anterior or posterior circulation, aneurysm size (<10mm vs ≥10mm), rebleeding, reintervention, early cerebral ischemia, hypertension, diabetes, hypothyroidism, depression, hypercholesterolemia, sepsis, shunting procedure, vasospasm, meningitis/ventriculitis, and diabetes insipidus.

**Table 3:** Significant predictors of early improvement in the univariate analysis.

**Variable**	**Odds ratio**	**95% Confidence interval**	**p**
Age ≤ 65 y	3.63	1.56–8.45	0.0020
WFNS I–II vs III–V	4.48	2.09–9.61	< 0.0001
Discharge mRS (1–2 vs 3–5)	3.55	1.64–7.68	0.00092
Absence of delayed cerebral ischemia	2.33	1.00–5.42	0.045
No history of smoking	0.44	0.20–0.95	0.035
Absence of pneumonia	3.05	1.45–6.42	0.0027
Absence of hydrocephalus	2.73	1.29–5.76	0.0075
Absence of tracheostomy	2.90	1.38–6.09	0.0041
No decompression	2.62	1.14–6.02	0.020
Time in ICU (≤ 3 weeks vs > 3 weeks)	2.46	1.18–5.12	0.015

Univariate analysis via chi-square test was performed to detect the significant predictors of early improvement from discharge to 6 months. n = 156 (1 missing).

### Predictors of late improvement in the univariate analysis

Predictors of late improvement were tested in the time interval from 6 months to 1 year. Chi-square testing was performed with the dichotomized data of 153 patients (n = 157; 4 missing at 1-year follow up). Early cerebral ischemia was associated with a late outcome improvement from 6 months to 1 year [odds ratio (OR) 0.41; p = 0.040]. The absence of a shunting procedure had a positive impact on the outcome improvement (OR 4.00; p = 0.021) (see Table 4). The following tested variables did not reach significance: early improvement (discharge to 6 months), intervention (clipping vs coiling), sex, age ≤ 65 y, WFNS I or II vs III, IV, or V, anterior or posterior circulation, aneurysm size, discharge mRS score (1–2 vs 3–5), rebleeding, retreatment, no DCI, hypertension, diabetes, hypothyroidism, depression, no history of smoking, hypercholesterolemia, absence of pneumonia, sepsis, absence of hydrocephalus, vasospasm, meningitis/ventriculitis, diabetes insipidus, tracheostomy, decompression, and time in ICU (≤ 3 weeks vs > 3 weeks).

**Table 4:** Significant predictors of late improvement in the univariate analysis.

**Variable**	**Odds ratio**	**95% Confidence interval**	**p**
Absence of early cerebral ischemia	0.41	0.17–0.97	0.040
No shunting procedure	4.00	1.14–14.05	0.021

Univariate analysis via chi-square test was performed to detect the significant predictors of late improvement from 6 months to 1 year. n = 153 (4 missing).

### Predictors of early improvement in the multivariate analysis

Logistic regression analysis was performed with the dichotomized improvement values of the mRSs from discharge vs the follow-up at 6 months (0 = “no improvement”; 1 = “improvement”) as the dependent variable. Improvement was defined as a decrease in the mRS from discharge to the 6-month follow-up of at least 1 mRS point. For this analysis, 156 of 157 cases were available (1 missing in the 6-month follow up). We used all 12 significant variables of the univariate analysis as covariates regarding the impact on the early improvement of the outcome. The Nagelkerke R-square value was 0.260, and the Hosmer–Lemeshow test value was 0.822. Age ≤ 65 y (OR 4.93; 95% CI 1.64–14.80; p = 0.0045) and a good WFNS grade (I or II) (OR 4.77; 95% CI 1.51–15.05; p = 0.0077) had a strong impact on the improvement of the outcome within the first 6 months after discharge (see Table 5).

**Table 5:** Predictors of early improvement in the multivariate analysis.

**Variable**	**Odds ratio**	**95% Confidence interval**	**p**
Age ≤ 65 y	4.93	1.64–14.80	0.0045
WFNS I–II vs III–V	4.77	1.51–15.05	0.0077
Discharge mRS (1–2 vs 3–5)	0.70	0.16–3.07	0.63
Absence of early cerebral ischemia	0.75	0.24–2.37	0.63
Absence of delayed cerebral ischemia	1.73	0.64–4.64	0.28
No history of smoking	0.87	0.34–2.17	0.76
Absence of pneumonia	1.17	0.31–4.37	0.81
Absence of hydrocephalus	1.64	0.59–4.55	0.34
No shunting procedure	0.63	0.19–2.08	0.45
No tracheostomy	0.73	0.15–3.57	0.70
No decompression	1.88	0.52–6.89	0.34
Time in ICU (≤ 3 weeks vs > 3 weeks)	1.38	0.50–3.85	0.53

Binary logistic regression was performed with dichotomized status of early mRS improvement from discharge to 6 months (0 = “no improvement”; 1 = “improvement”) as the dependent variable and age, WFNS grade, discharge mRS, early cerebral ischemia, delayed cerebral ischemia, history of smoking, pneumonia, hydrocephalus, shunting procedure, tracheostomy, decompression, and time in ICU as independent variables (covariates). n = 156 (1 missing).

### Predictors of late improvement in the multivariate analysis

For the testing of predictors of late improvement (6 months to 1 year) in the multivariate analysis, we also performed a logistic regression analysis with the 12 significant variables of the univariate testing as the dependent variables (covariates). The improvement definition and covariate usage were the same as in the multivariate analysis of early improvement. For this analysis, 153 of 157 cases were available (4 missing in the 1-year follow up). The Nagelkerke R-Square value was 0.187, and the Hosmer–Lemeshow test value was 0.801. Age ≤ 65 y and a good WFNS grade had no significant impact on the late improvement of the outcome (see Table 6). Instead, the absence of a shunting procedure became a significant strong predictor for the improvement of late outcome (OR 8.32; 95% CI 1.91–36.34; p = 0.0049).

**Table 6:** Predictors of late improvement in the multivariate analysis.

**Variable**	**Odds ratio**	**95% Confidence interval**	**p**
Age ≤ 65 y	0.72	0.25–2.081	0.54
WFNS I–II vs III–V	1.06	0.37–3.059	0.92
Discharge mRS (1–2 vs 3–5)	0.31	0.087–1.11	0.072
Absence of early cerebral ischemia	0.52	0.19–1.48	0.22
Absence of delayed cerebral ischemia	1.57	0.55–4.43	0.40
No history of smoking	0.74	0.32–1.67	0.46
Absence of pneumonia	2.58	0.72–9.27	0.15
Absence of hydrocephalus	0.48	0.20–1.18	0.11
No shunting procedure	8.32	1.91–36.34	0.0049
No tracheostomy	0.67	0.14–3.34	0.63
No decompression	1.12	0.27–4.69	0.88
Time in ICU (≤ 3 weeks vs > 3 weeks)	1.25	0.46–3.39	0.67

Binary logistic regression was performed with dichotomized status of late mRS improvement from 6 months to 1 year (0 = “no improvement”; 1 = “improvement”) as the dependent variable and age, WFNS grade, discharge mRS, early cerebral ischemia, delayed cerebral ischemia, history of smoking, pneumonia, hydrocephalus, shunting procedure, tracheostomy, decompression, and time in ICU as independent variables (covariates). n = 153 (4 missing).

## DISCUSSION

In our study, the majority of improvement after SAH occurred within the time interval of discharge to 6 months (mRS at discharge vs mRS at 6 months: 3.32 +/- 1.91 vs 2.59 +/- 2.37; p < 0.0001), but also the time interval from 6 months to 1 year played a significant role in the recovery process (mRS at 6 months vs mRS at 1 year: 2.58 +/- 2.38 vs 2.36 +/- 2.55; p = 0.00077). Every forth to fifth patient with potential of recovery (mRS 1-5) at discharge improved by 1 mRS point in the time interval from 6 month to 1 year (22.6%). Patients with mRS 3 at discharge had a remarkable late recovery rate (73.3%, p = 0.000085). We detected age ≤ 65 years (OR 4.93; p = 0.0045) and WFNS grade I or II (OR 4.77; p = 0.0077) to be significant predictors of early improvement (discharge to 6 months) in our multivariate analysis. Chronic hydrocephalus followed by a shunting procedure represents a significant risk factor against late improvement after SAH (OR 8.32; p = 0.0049) in the analyzed collective, but not age ≤ 65 years and WFNS grade I and II.

### Dynamic of recovery after SAH

Recovery after SAH is a dynamic process, and late (after 6 months) improvement of the patients should not be underestimated [1, 4]. Motor and psychomotor recovery seems to occur mainly within the first 6 months after aneurysmal SAH, but other cognitive aspects—such as verbal memory—may need longer recovery time [12]. Hop et al. saw an improvement between 4 months and 18 months post-SAH (at least one point on the mRS) in 50% of their collective [13]. However, until 5 years post-SAH, the recovery continued only slightly (56%). At 12.5 years, no further improvement was detectable [14]. Das et al. retrospectively analyzed a cohort of 85 poor-grade (Hunt and Hess 4 and 5) patients suffering from aneurysmal SAH who were treated by microsurgical clipping. In their study, functional improvement was steady with time, although certain complications interrupted the recovery process between 6 and 18 months [1]. Marked improvement occurred in 11% of the survivors even after 18 months. Therefore, they stated that outcome assessment and further rehabilitation efforts are important also for longer time intervals [1]. In the partly prospective, partly retrospective study of Wilson et al., 88 cases of poor-grade aneurysmal SAH were analyzed. They examined the collective from discharge to 6 months and found 61% improvement of at least one mRS grade, which is quite similar to our findings (57.4%), as well as regarding the time interval between 6 months and 1 year (18% improvement of the collective in Wilson et al. vs 22.6% in our study) [4]. They reported the mean aggregate mRSs at 6 months (3.31 ± 2.1), 12 months (3.28 ± 2.2), and 36 months (3.17 ± 2.3) and detected a significant improvement compared with the mean score at hospital discharge (4.33 ± 1.3, p < 0.001).

However, among the particular time-intervals, there was no significant difference. In our study, we analyzed a full collective of SAH patients with the range of WFNS I to V in order to get a more generalized overview regarding potential outcome improvement factors after SAH. The mRS improvement from discharge to 6 months was a bit higher in the study of Wilson et al. (mRS improvement 1.02) compared to our results (mRS improvement 0.73) [4]. For the time interval from 6 months to 1 year, the mean aggregate mRSs were 3.31 and 3.28, with a nonsignificant improvement of only 0.03 mRS points. This contrasts with our results, in which we detected a significant (p = 0.00077) improvement of 0.22 mRS points (2.58 at 6 months and 2.36 at 1 year). Wilson et al. emphasized the importance of the late recovery after SAH, but they could not argue with the mean aggregate mRS improvement after 6 months, because it was insignificant. Thus, they highlighted the individual recovery of the patients. This was the main point of critique, as individual patient outcomes are not the point in clinical trials [3–5]. Moreover, the small number of patients and the subset of patients with a Hunt and Hess grade of IV and V were criticized. With our finding of statistical significance of the mean aggregate mRS improvement from 6 months to 1 year in a larger and more generalized SAH collective, the argument of unimportance of this time interval seems to be obsolete [3]. It underscores the importance of the time up to 1 year after discharge for the recovery of the patients, even without a closer look at their individual courses of recovery [4].

Figure 2 shows the recovery for each mRS class and each time interval. Recovery occurs especially up to 6 months for patients with mRS 1-3 at discharge (mRS 1: 32 of 37, 86.5%; mRS 2: 38 of 46, 82.6%, 1 missing; mRS 3: 13 of 15, 86.7%). Almost all patients in this class improved within the first 6 months. From 6 months to 1 year there is a reasonable individual recovery rate, which can be observed for patients with mRS 2 (17 of 46, 37.0 %, 1 missing), but not for mRS 1 patients (mRS 1: 3 of 37, 8.1%; significantly lower than mRS 2-5 in the chi square testing: p = 0.0011). Interesting is the late recovery rate of mRS 3 patients, where 11 of 15 patients improved (73.3%, significantly higher than mRS 1-2 and 4-5 in the chi square testing: p = 0.000085). Patients with mRS 4 have an acceptable recovery rate from discharge to 6 months (mRS 4: 20 of 31, 64.5%). Patients with mRS 5 have a significant lower early recovery rate than the rest of the collective with mRS 1-4 (mRS 5: 13 of 28, 46.4%, chi square testing: p= 0.00019). From 6 months to 1 year the recovery rate of mRS 4 patients is comparable to the respective recovery rate of mRS 2 patients (mRS 4: 9 of 31, 29.3%, 1 missing). The late recovery rate of mRS 5 patients is moderate (5 of 28, 17.9%, 2 missing).

### Predictors of recovery after SAH

Wilson et al. also analyzed their collective with univariate statistical methods in order to detect predictors of early and delayed neurological improvement [4]. Factors predicting the improvement from discharge to 6 months were a better Hunt and Hess Grade (IV vs V: OR 6.20; 95% CI 2.11–18.25; p < 0.001) and the absence of large (> 4 cm) (OR 2.76; 95% CI 1.02–7.55; p = 0.05) or eloquent (OR 5.17; 95% CI 1.89–14.10; p < 0.01) stroke. For the time interval from 6 months to 1 year, age ≤ 65 years (OR 5.56; 95% CI 1.17–26.42; p = 0.02), a better Hunt and Hess Grade (IV vs V: OR 4.17; 95% CI 1.10–15.85; p = 0.03), and absence of a large (OR 8.97; 95% CI 2.65–30.40; p < 0.001) or eloquent (OR 4.54; 95% CI 1.46–14.08; p = 0.01) stroke were associated with improvement [4]. In our study, the WFNS Grade (I–II vs III–V: OR 4.48; 95% CI 2.09–9.61; p < 0.0001) and the absence of a stroke (absence of DCI but not ECI; absence of DCI: OR 2.33; 95% CI 1.00–5.42; p = 0.045) also played an important role in the prediction of early outcome improvement in the univariate analysis between several more factors, such as age ≤ 65 yrs, discharge mRS, no history of smoking, absence of pneumonia, absence of hydrocephalus, absence of tracheostomy, no decompression, and time in ICU ≤ 3 weeks. For the time interval from 6 months to 1 year, the early cerebral ischemia (absence of early cerebral ischemia: OR 0.41; 95% CI 0.17–0.97; p = 0.040), but not age ≤ 65 years or the WFNS grade, was significant. Recovery from ischemic stroke as part of the SAH disease or in terms of procedure-related complications, which might be more circumscribed than DCI, could be the reason for this result in the univariate analysis. Ganesh et al. detected late recovery in ischemic stroke patients in 25% of the patients. A mRS improvement by ≥1 point from 3 months to 1 year occurred in 317 of 1266 patients with 3-month mRS ≥1 [15]. Ganesh et al. also detected, that some subtypes of stroke, like lacunar stroke, are more likely to demonstrate late improvement between 3 months and 1 year with multivariate analysis methods [16]. However, we could not confirm this late recovery effect for early cerebral ischemia in our multivariate analysis (see Table 6) (Absence of early cerebral ischemia OR = 0.52; 95% CI = 0.19–1.48; p = 0.22). Moreover, the absence of a shunting procedure was a significant factor for late improvement (OR 4.00; 95% CI 1.14–14.05; p = 0.021) in the univariate analysis.

Another criticism regarding the paper of Wilson et al. was the lack of multivariate analysis in order to detect independent information [3, 4]. In our multivariate analysis, we found the WFNS grade to be a significant predictor for improvement from discharge to 6 months (WFNS I–II vs III–V: OR 4.77; 95% CI 1.51–15.05; p = 0.0077), in addition to age ≤ 65 y (OR 4.93; 95% CI 1.64–14.80; p = 0.0045). However, neither ECI nor DCI played a significant role for this period (ECI: p = 0.63; DCI: p = 0.28). For the multivariate analysis of the period from 6 months to 1 year, none of the factors Wilson et al. found could be confirmed [4]. Only the absence of a shunting procedure was a very strong predictor for late improvement (OR 8.32; 95% CI 1.91–36.34; p = 0.0049).

### Late improvement is independent from age and WFNS grade

Demographic shift towards a large proportion of old patients leads to increasing demand on healthcare. It is known that patients above 80 years suffering from ischemic stroke, have a higher risk-adjusted fatality, longer hospitalization, and were less likely to be discharged to their original place of residence. Strategies need to be inducted in order to encounter this challenge [17]. Poor and unspecialized management of elderly patients suffering from stroke might even lead to higher financial and healthcare burden in the form of increased morbidity, long term disability, and placement in residential care [18]. Goldberg et al. found in a collective of poor grade patients that unfavourable outcomes 6 to 12 months after aneurysmal SAH were strongly related to older age. However, treatment of patients up to 79 years resulted in a considerable proportion of favourable outcomes and only a small number of patients who were moderately or severely disabled 6 to 12 months after aneurysmal SAH [19].

In our study we found a reasonable and significant improvement of outcome from 6 months to 1 year which was independent from age and WFNS grade (see Tables 2 and 6). This is in contrast to the period from discharge to 6 months, where age and WFNS grade played a significant role regarding the recovery process (see Table 5). The first months after aneurysmal SAH are critical especially for older patients and poor WFNS grade regarding complications after SAH. Late occurring complications like shunt dysfunction or interactions with comorbidities might occur. The time interval from 6 months to 1 year describes a more rigid space of time, where on the one hand the total recovery is not as high as in the initial time interval, but on the other hand it is a more stable phase, where critical late complications after SAH are scarcely to be expected. Wilson et al. did not detect this significant recovery in the time interval from 6 months to 1 year. Moreover, a dependency of the late recovery on age and hunt and hess grade was found. Reasons for this issue might be the considerably smaller collective, univariate analysis methods and the restriction on poor grade patients [4].

There are multiple reasons, why we chose “age less or equal 65 years” as the border for our dichotomization. We wanted to make our results to be comparable to other data, especially to the data of Wilson et al. Moreover, there was a need to define a limit where patients are considered to be “old”. This stage comes along with opinions of restrictive treatment regarding intensive care and neurovascular approaches in the daily clinical routine. These patients are considered to have limited ability to overcome severe diseases and limited potential of recovery. There is a need to (re-)evaluate these borders in terms of new treatment methods and improving health over time regarding SAH.

Additionally, we performed the same regression analyses as showed in Tables 5 and 6 with age ≤ 50, 55 and 60 years as borders for old age. For all regression analyses the significant predictors / risk factors remained significant. There were no additional significant predictors within these regression analyses, especially regarding late recovery considering “age” and “WFNS grade”.

### Limitations

Regarding comorbidities and history of smoking, the data were collected on patients’ admissions and were documented in the patients’ files. However, analysis of this data for the purpose of this study was done retrospectively [9]. Parts of the data regarding the intensive care complications and interventions were collected retrospectively. No power analysis for definition of the case number had been performed. Acquisition of follow-up data was done by telephone interview, which is potentially less reliable than physical neurological examinations, as stated before [9].

There is a strong association of the volume of SAH cases treated with the outcome. The definition of high volume centers differ from 20 cases per year (limit for obtaining Comprehensive Stroke Center (CSC) certification) to 100 cases per year [20–22]. Rush et al. detected, that after adjustment for all baseline covariates, including severity of SAH, treatment in a high-volume center defined with 20 SAH cases per year was associated with a decreasing odds ratio for death and a higher odds of a good functional outcome [21].

Pandey et al. examined the impact of the SAH volume per year and found, that even a higher SAH volume is critical regarding mortality and discharge home. As SAH volume decreased from 100/year, mortality increased steadily from 18.7% (100 cases per year) to 28.4% (20/year). Moreover, discharge home was more likely with increasing SAH volume (40.3% at 100/year to 35.3% at 20/year) [22].

With approximately 50 SAH cases per year we treat an amount of SAH cases which lies between the minimum criteria of 20 cases per year and the maximum criteria of 100 cases per year.

The total number of cases and the number of cases of each independent variable was moderate, which makes selection effects very pronounced and could leave relevant summation effects of different variables undervalued. Over 80.9% of the patients were ≤ 65 years old and 61.8% had a very good to good initial clinical condition (WFNS grade I+II). With a larger collective, additional statistical methods (like the AUC calculation) would have come to consideration, which might give additional information and information of more value. More powerful prospective randomized multicenter trials with high patient numbers will be needed to confirm our results.

## CONCLUSIONS

Recovery after SAH is a dynamic process, with significant improvement beyond 6 months—not only regarding individual recovery, but also regarding the mean aggregate mRS. Every forth to fifth patient with potential of recovery at discharge improved by 1 mRS point in the time interval from 6 month to 1 year (22.6%). While age and WFNS grade are the driving factors for early improvement, chronic hydrocephalus with consecutive shunting procedures is a severe risk factor against potential late improvement after SAH. Outcome improvement from 6 month to 1 year is significant and independent from age and WFNS grade. Patients with mRS 3 at discharge had a remarkable late recovery rate. In these terms, individualized and prolonged intensive long-term rehabilitation is an important factor in the recovery process even for high grade patients and patients above 65 years suffering from aneurysmal SAH.

## MATERIALS AND METHODS

Data from 203 cases of SAH were extracted from our observational database from the years 2012 to 2017.

Patients were included if the time between aneurysm rupture and treatment was <48 hours, if informed consent was given by the patient, a patient’s relative, or the patient’s guardian, if the SAH was detected via cranial CT or lumbar puncture, if an associated intracranial aneurysm was discovered by digital subtraction angiography or by CT angiography, and if the patient survived until completion of the aneurysm treatment. In terms of the standard care, specialized neurosurgeons and endovascular specialists assigned the patient to the endovascular group or the clipping modality group and executed treatment for occlusion of the ruptured aneurysm. In the endovascular group, sole coiling, coiling in combination with balloon- or stent-assisted remodelling, or the use of endovascular or intrasaccular flow diverters were used.

Functional outcomes were classified using the modified Rankin Score (mRS), which was prospectively determined at discharge, then at 6 months and 1 year by telephone interview. The telephone interviews were constantly performed by the same physician of the department of neurosurgery. Informed consent of the patients or their relatives was obtained during the initial hospital stay or during the telephone interview in the follow-up. The study was approved by the local review board (Ethics Committee of the Bavarian Chamber of Physicians; 2019-124 Dr. AB/Gu).

We recruited cases of SAH from 2012 to the early year of 2016. After that the telephone interviews were performed until the early year of 2017. Moreover 46 patients suffered from SAH without detection of an aneurysm and one patient refused to participate in this study. In 4 years, we collected 203 cases for the SAH-database. In 4 years of recruitment an average of approximately 50 patients were treated per year.

There were two major aspects to this study: comparison of the mean aggregate mRSs at different time points and predictors of improvement in the mRS over time. For the comparison of the mean aggregate mRSs, we regarded the entire collective of 203 patients. One patient was missing at the 6-month follow-up, and 4 patients were lost at the 1-year follow up due to missing accessibility regarding the known contact data. Therefore, for the half-year analysis there were 202 patients, and for the 1-year follow-up 199 patients were available. In terms of the analysis of predictors of improvement of the mRS, we excluded patients with an mRS of 6 at discharge (deceased, n = 39) and patients that had an excellent mRS of 0 at discharge (n = 7). No patient with an mRS of 0 at discharge had deteriorated at the 6-month or 1-year follow-up. This was done in order to analyze the dynamic data exclusively. Accordingly, 157 patients remained for the analysis of the dynamics of outcome. Again, 1 patient was missing at the half-year follow-up (n = 156) and 4 patients in the 1-year follow-up (n = 153).

We categorized outcome measures as “improvement of clinical outcome” (at least 1 point in the mRS) or “no improvement” (static mRS or a decline in the mRS) over time.

Early cerebral ischemia was defined as a clinically apparent new ischemia within the first 3 days after treatment, detectable on cranial computed tomography scan via new hypodensities compared to the cranial computed tomography scan at admission. Potential small infarcts without detection in the cranial CT scan and without apparent new neurological deficit were not detected / recorded. We made no precise distinction between a periprocedural ECI or ECI caused by SAH. All clinical events that occurred 3 or more days after treatment were also confirmed via new hypodensities in a cranial CT scan until discharge and were defined as delayed cerebral ischemia (DCI), which included a focal (hemiparesis, aphasia, hemianopia, or neglect) or global neurological impairment lasting at least 1 hour, and/or cerebral infarction, not apparent immediately after aneurysm treatment and not attributable to other causes [8].

We partially used the data of this study for previous publications with different objectives [9–11, 23]. Regarding which variables might influence outcome, we used the available respective baseline, infarction, comorbidity, smoking, and ICU complications/interventions data as partially described before [9]. Data concerning pre-defined comorbidities and history of smoking were surveyed on the patient’s admission and documented in the patient’s file. However, the analysis of this data, for the purpose of this study, was done retrospectively [9, 23]. Data concerning the intensive care complications and interventions were collected during the stay in the intensive care unit by personal communication with the treating intensive care physicians and a search of the available medical records.

### Statistics

Statistical analysis was performed using SPSS version 21 software (SPSS Inc., Cary, SC). For the examination of the mean aggregate mRS at discharge, 6 months, and 1 year, we used the paired samples t-test. Regarding the investigation of significant mRS values at discharge for early and late improvement we used a chi-square test. Also, for the analysis of existing predictors of outcome improvement, we used a chi-square test in order to filter our primary variables regarding covariates for the multivariate analysis (binary logistic regression). Therefore, only the significant variables of the univariate testing were used as independent variables (covariates) in the multivariate analysis. This was in line with a possible pathophysiological hypothesis defined by the authors. We included a maximum of 12 independent variables in the logistic regression. The number of cases of each independent variable was ≥ 26. The Nagelkerke R-square and Hosmer–Lemeshow test values were stated along with the executed binary regression analysis. P-values < 0.05 indicated statistical significance.
